# Soft-bottom fishes and spatial protection: findings from a temperate marine protected area

**DOI:** 10.7717/peerj.4653

**Published:** 2018-06-08

**Authors:** Inês Sousa, Jorge M.S. Gonçalves, Joachim Claudet, Rui Coelho, Emanuel J. Gonçalves, Karim Erzini

**Affiliations:** 1 Centre of Marine Sciences—CCMAR, University of Algarve, Faro, Portugal; 2 National Center for Scientific Research, PSL Université Paris, CRIOBE, USR 3278 CNRS-EPHE-UPVD, Maison des Océans, Paris, France; 3 Laboratoire d’Excellence CORAIL, France; 4 Instituto Português do Mar e da Atmosfera—IPMA, Olhão, Portugal; 5 MARE—Marine and Environmental Sciences Centre, ISPA—Instituto Universitário, Lisboa, Portugal

**Keywords:** Soft-bottom fishes, Marine protected areas, Reserve effect, Multivariate regression trees, Catch per unit effort

## Abstract

Numerous studies over the last decades have focused on marine protected areas (MPAs) and their effects on fish communities. However, there is a knowledge gap regarding how species that live associated with soft-substrates (e.g., sand, mud) respond to spatial protection. We analyzed abundance, biomass and total lengths of the soft-bottom fishes in a multiple-use MPA in the north-eastern Atlantic, the Luiz Saldanha Marine Park (Portugal), during and after the implementation of its management plan. Data were collected by experimental fishing in areas with three different levels of protection, during the implementation period and for three years after full implementation of the MPA. Univariate analysis detected significant biomass increases between the two periods. Fish assemblages were mainly structured by depth and substrate, followed by protection level. Community composition analyses revealed significant differences between protection levels and between the two periods. Species exhibited a broad variation in their response to protection, and we hypothesize that factors such as species habitat preferences, body size and late maturity might be underlying determinants. Overall, this study provides some evidence of protection effectiveness in soft-bottom fish communities, supported by the significant increase in biomass in the protected areas and the positive trends of some species.

## Introduction

The marine environment is nowadays increasingly impacted by human activities, with overfishing reported worldwide (Myers & Worm, 2003; Pauly & Palomares, 2005). As a consequence, conservation of marine fishes currently holds unique challenges for fisheries managers (Vincent & Hall, 1996; Roberts, 1997). Marine protected areas (MPAs) are one of the key tools to implement ecosystem-based management and marine spatial planning (Gaines et al., 2010; Halpern, Lester & McLeod, 2010). As areas with restrictions to human uses, MPAs can promote the preservation and recovery of habitats and marine species, and are increasingly being used as tools for conservation and fisheries management (Roberts & Polunin, 1993; Gaines et al., 2010). Through the reduction of fishing mortality, these areas can enhance density, biomass, fish size and species diversity within their boundaries (Osenberg et al., 2011), which is often termed the ‘reserve effect’. Moreover, the transport of larvae (‘recruitment subsidy’) and movement of juveniles and adults (‘spillover’) to the outside areas are expected to increase fisheries yields (Gell & Roberts, 2003; Grüss et al., 2011).

MPAs include marine reserves or fully protected areas (FPAs), where extractive activities are prohibited (no-take zones), and partially protected areas (PPAs), which allow multiple uses (Lubchenco et al., 2003). PPAs offer a broad spectrum of protection levels, allowing either many human activities or just a few and like no-take zones, they have the potential to enhance social and ecological benefits in coastal areas (Guidetti & Claudet, 2010; Claudet & Guidetti, 2010; Sciberras et al., 2013; Sciberras et al., 2015; Horta e Costa et al., 2016).

For the achievement of conservation and management goals, it is important to understand the ecological effects of MPAs and the factors underlying these effects. The effectiveness of MPAs for the recovery of fish communities is affected by spatial design (Halpern & Warner, 2002; Claudet et al., 2008), enforcement (Byers & Noonburg, 2007; Campbell et al., 2012; Bergseth, Russ & Cinner, 2015), habitat heterogeneity (García-Charton & Pérez-Ruzafa, 1999), species movement ecology (Chapman et al., 2005; Villegas-Ríos, Moland & Olsen, 2017) and life history traits (Jennings, Greenstreet & Reynolds, 1999; Claudet et al., 2010; Hutchings et al., 2012). Life history traits, such as longevity, maximum size, growth rate and reproductive potential, are important components of species responses to environmental stressors (e.g., temperature, toxicants, food availability) and fishing exploitation (Jennings, Greenstreet & Reynolds, 1999; Denney, Jennings & Reynolds, 2002; Hutchings et al., 2012; Audzijonyte & Kuparinen, 2016). Species habitat preferences and inter-specific relations are acknowledged as important sources of complexity in spatial protection effectiveness, alongside with movement ecology (García-Charton & Pérez-Ruzafa, 1999; Claudet et al., 2010). The fact that there is still no clear consensus on the influence of migratory behavior as a determinant of protection effectiveness (Blyth-Skyrme et al., 2006; Claudet et al., 2010) illustrates this complexity.

Despite the challenges posed by the study of spatial management, MPAs have been shown to enhance the recovery and resilience of rocky (Claudet et al., 2006; Tetreault & Ambrose, 2007; Sala et al., 2012) and coral reef fish (Russ & Alcala, 2004; Bruce et al., 2012). However, there has been relatively little research on the response to protection of soft-bottom fish communities, probably because most MPAs target reef-associated species and because standard survey methods such as underwater visual census are regarded as not suitable for soft-bottom habitats, where fish densities are generally low (Giakoumi & Kokkoris, 2013).

Some MPA assessments have included multiple soft-bottom fish species among extensive community studies (e.g., Dimech et al., 2008; Guidetti & Claudet, 2010), and others addressed single soft-bottom species (e.g., Hunter et al., 2006; Wiegand, Hunter & Dulvy, 2011; Silva et al., 2013; Abecasis, Afonso & Erzini, 2014). Piet & Rijnsdorp (1998) reported that the ‘plaice box’, an area in the North Sea with seasonal fishing restrictions, favored the occurrence of larger fish. In the Western Atlantic, Georges Bank and southern New England fishing closures proved beneficial to several species of flounders and skates (Murawski et al., 2000), and significant increases in the abundance of winter flounder (*Pseudopleuronectes americanus*) and other fishes were reported after implementation of a trawling closure on the Scotian Shelf (Fisher & Frank, 2002). Pipitone et al. (2000) also described increases in the abundance and biomass of some soft-bottom fishes (e.g., *Mullus barbatus* and *Lepidotrigla cavillone*) after a trawling ban in a Mediterranean MPA. More recently, Donovan et al. (2016) reported the effects of a gillnet ban in a sandy coastal area of Hawai‘i, and highlighted the variability of response according to species group, as only bonefishes (*Albula* spp.) exhibited a positive trend.

In this study we assessed the response to protection at the community and species level of the soft-bottom fish assemblage in the Luiz Saldanha Marine Park (LSMP), a coastal MPA in Portugal with predominantly soft-substrates (Henriques et al., 2014). Data were collected using standardized fishing procedures (trammel nets) inside and outside the marine reserve, over the implementation period and after the MPA was fully established. We used univariate and multivariate methods to analyze trends in community composition, fish abundance and biomass, and mean total length per species according to protection level, from before to after the full implementation of the MPA.

## Materials & Methods

### Study area

Located on the western coast of Portugal, the Luiz Saldanha Marine Park (LSMP) comprises 38 km of coastline characterized by high rocky cliffs alternating with sheltered bays. With an area of 53 km^2^, the park includes many sublittoral habitat types, including hard and soft substrates. Soft bottoms replace the coastal rocky reefs in most of the area at depths greater than 15–20 m. A substantial mud component is found in the sediment at depths greater than 30 m, while at shallower depths the dominant substrate is sand (medium, coarse and gravely sand) (Henriques et al., 2014). One interesting aspect to point out is the wide variety of soft-substrate habitats and benthic communities found in this area, as described by Henriques et al. (2014). The area has two nearby estuaries (Sado and Tejo) and is next to the Arrábida mountain chain, which shelters it from the dominant north winds. This region is also a biogeographic and oceanographic transition zone between warm and cold temperate waters (Henriques, Gonçalves & Almada, 1999; Lima et al., 2007) which, along with the variety of available habitats (Henriques et al., 2014), makes it an important biodiversity hotspot (Henriques, Gonçalves & Almada, 1999; Gonçalves, Henriques & Almada, 2002).

**Figure 1:**
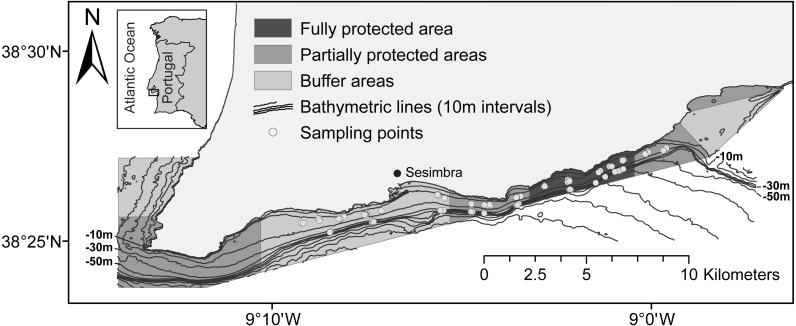
Map of the Luiz Saldanha Marine Park (LSMP) zoning. The light grey dots indicate sampling points (experimental fishing).

The MPA was created in 1998 as part of the Arrábida Natural Park, but it was only in August 2005 that the management plan started to be implemented. The management regulation main goals are to enhance the conservation of marine species and promote sustainable fisheries management (Gonçalves, Henriques & Almada, 2002). Given the diversity of human activities, including recreational fishing, scuba diving and commercial fisheries, and the presence of two important fishing harbours: the port of Sesimbra, located within the MPA area, and the nearby port of Setúbal, spatial management was crucial. To mitigate conflicts related to multiple stakeholders and their divergent concerns (Vasconcelos et al., 2013), the LSMP regulations were established gradually over a four-year period and full implementation was achieved in August 2009 (implementation: 23rd Aug. 2005–23rd Aug. 2009; Year 1: LSMP area under buffer area protection level; Year 2: two PPAs were implemented; Year 3: two more PPAs were created, sampling started; Year 4: half of the FPA implemented; 23rd Aug. 2009—the FPA is fully implemented). The regulations define different zones and levels of usage (Fig. 1). Trawling, dredging, purse seining and spearfishing are prohibited in the entire area, and only licensed commercial vessels less than 7 m long are allowed. The fully protected area (FPA) (4 km^2^) excludes all human activities, with the exception of research and monitoring. The four partially protected areas (PPA), totalling 21 km^2^, allow some recreational activities, including recreational diving, and commercial fishing for cephalopods with octopus traps and jigs, although fishing is not allowed at distances less than 200 m from the shore. The three buffer areas (BA), totalling 28 km^2^, have the lowest level of protection. In addition to octopus traps and jigs (here permitted close to the shore), longlines, trammel nets, gill nets and recreational fishing are allowed (nets at a distance beyond 463 m from the shoreline).

### Data collection

Data were collected between November 2007 and May 2012 over a five years period: the first two years during the management plan implementation (period 1—implementation period: August 2007–August 2009) and three years after the MPA was fully implemented (period 2—after MPA full implementation: August 2009–August 2012). Sampling of the three protection levels occurred seasonally, each spring and autumn. Ten sampling campaigns (experimental fishing) and 183 trammel net sets were undertaken (Fig. 1) (total number of samples: 179; four samples were excluded from the analysis due to problems with the fishing procedure). Sampling area covered the FPA (*n* = 63), two adjacent PPAs (*n* = 61) and the larger BA (*n* = 59). To avoid spatial heterogeneity, areas in the eastern and western edges of the MPA were not included (west: proximity to cape; east: proximity to nearby estuary). Four sampling campaigns were undertaken during period 1 (*n* = 57) and six during period 2 (*n* = 126) (first three campaigns—four days each, 36 samples; following campaigns—seven days each, total of 147 samples; three samples per day). Two substrate types were sampled: sandy bottom, at 10–20 m (*n* = 112), and muddy bottom, at 30–45 m (*n* = 71) (samples: sand-BA: 36, PPA: 38, FPA: 38; mud-BA: 23, PPA: 23, FPA: 25). The BA was considered as the control in the spatial analysis of protection effect due to the allowed use of small-scale fishing gear: gill nets, trammel nets, longlines, jigs, pots and traps. The area surrounding the MPA was not chosen as a control because of greater depths and differences in bottom type. Each sample consisted of the catch of one monofilament trammel net (500 m long; 1.6 m high; inner panel stretched mesh of 100 mm; outer panel stretched mesh of 600 mm; soak time 22–24 h). Nets were set after sunrise and hauled about 24 h later. Each fish was identified on board to the species level and total length (TL) was measured to the nearest millimetre. Specimens were released afterwards, following ‘catch and release’ practice (The Portuguese Institute for Nature Conservation and Forests—ICNF approved the field surveys for this study; ICNF reference no 00540 140307). Biomass was estimated using published length-weight relations (Table S1—list of species with length-weight references, environment, maximum size and fisheries category). Catch in number per unit effort (CPUE) and biomass per unit effort (BPUE) were calculated considering the unit effort as 500 m of trammel net and a soak time of 24 h. This provided average values (abundance and biomass) per sample that enabled comparisons of the three protection levels over time.

### Data analysis

CPUE expressed as number of fish per 500 m of net (n⋅500 m^−1^) was considered as an index of relative abundance and used along with biomass (BPUE—kg⋅500 m^−1^) to evaluate differences between protection levels and trends over time (before and after full MPA implementation). Prior to data analysis, pelagic species (*Auxis rochei*, *Boops boops*, *Sarda sarda*, *Sardina pilchardus*, *Scomber* spp*.*, *Trachurus* spp.) were excluded from the dataset. Pelagic species are expected to be less meaningful in habitat characterization (Fréon & Misund, 1999) and therefore were not analyzed.

We used generalized linear mixed models (GLMM) (Breslow & Clayton, 1993) to test significance of protection level (BA, PPA, FPA), substrate (sand, mud) and period (period 1, period 2) as predictors of abundance and biomass. The interaction of protection level and substrate was included in the models to assess whether the effect of protection level was different in each substrate type. Sampling campaign (*n* = 10) was included as random variable to deal with correlation between observations and with the variability related with campaigns (Zuur et al., 2009). Models were fitted by maximum likelihood with Laplace approximation. Poisson and Gamma distributions (log link) were used to fit, respectively, abundance (discrete) and biomass (continuous) data. Variable selection was carried out by likelihood ratio tests between nested models and by looking to the deviance explained by each variable, while model validation was carried out with visual inspection of the residuals (Zuur et al., 2009).

To analyse species spatial and temporal patterns, ratios of abundance, biomass and total length per species (for stingrays—*Myliobatis aquila*—dimension used was disc width) were also estimated and applied in accordance with a Before-After-Control-Impact (BACI) analysis approach (Underwood, 1992; Underwood, 1994). Comparisons were made between the buffer area (BA) and the two higher protection levels combined (PPA and FPA: PA—protected area), and between period 1 (implementation period) and period 2 (after full implementation) (protection level ratios included data from both periods; period ratios included data from the three protection levels). This specific analysis was done for the species with frequency of occurrence higher than 15% (Table S2). The average ratios (abundance, biomass and total length), standard errors (SE) and 95% confidence intervals (CI) were obtained by bootstrapping (9,999 permutations). The 95% confidence intervals were calculated using the adjusted bootstrap percentile method and used to check for significance (Payton, Greenstone & Schenker, 2003). To avoid problems resulting from small sample size, bootstrapping of total length was only performed for species with more than ten individuals per treatment (protection level or period). Hence, for total length comparisons, the species *Citharus linguatula*, *Raja undulata* and *Solea solea* were excluded from the protection level ratios, and *C. linguatula*, *R. undulata* and *Rostroraja alba* were excluded from the period ratios.

Multivariate procedures were applied to examine differences in fish assemblage according to protection level (PL) and time (period and year). The Hellinger transformation was used for abundance and biomass in order to overcome the problem of zero inflation (Legendre & Gallagher, 2001). Permutational multivariate analysis of variance (PERMANOVA) (Anderson, 2001) was performed to test significance of protection level, period and year as factors for community differences. Each constraint factor was assessed individually and then under interaction models: ‘protection level × period’ and ‘protection level × year’. Pairwise analyses were also obtained by comparing the protection levels within each period and comparing periods of each protection level.

The Euclidean distance matrix of transformed abundances was used in a principal components analysis (PCA). This technique provides visualization of multivariate data structure. It was used to explore the similarity between samples according to protection level and period. In order to compare samples, centroids per protection level and period, and dispersion ordiellipses (95% confidence interval) according to the weighted average scores of each protection level, were plotted. A second graphical output was also obtained with the species vectors in order to assess species contributions for community differences. Given the similarity of results obtained for abundance and biomass, the multivariate visualizations based on biomass are not presented.

To explore the main factors structuring fish assemblages, multivariate regression tree (MRT) analysis (De’Ath, 2002) was carried out using the Hellinger transformed abundances. The factors ‘protection level’ and ‘period’, and two environmental variables—depth and temperature were included. Substrate type was not included because it is correlated with depth, and priority was given to the variable that incorporated more information (depth). This analysis creates clusters of sites by splitting the data according to explanatory variable threshold values and site similarity. The clusters and their dependence on the environmental data are represented graphically by a tree. Through this procedure, each cluster defines a species assemblage, and its environment type is described by the associated environmental values. MRT was performed given its ability to deal with discrete variables (in our case, protection level and period), non-linearity, and higher-order interactions among explanatory variables (De’Ath, 2002). The criterion for final model selection was minimization of cross-validated relative error (CVRE).

Indicator values (IndVal) (Dufrêne & Legendre, 1997) were calculated for each species in each node and terminal node (leaf). With this index, species are considered as indicators of a certain cluster of sites according to their relative abundance and relative frequency of occurrence within that group. Specifically, the product of relative abundance and relative frequency of occurrence of the species within a group is calculated and then multiplied by 100. The index takes the value of zero if there is no occurrences of the species within a group, increasing to 100 if the species occurs at all sites within a group and does not occur in any other group. A minimum of 10% contribution to the explained variance was required for a species to be considered as discriminant at a particular node and permutations were used to test for significance. Permutations were also used to assign significant discriminant species for each terminal node. A species was considered an indicator of a certain assemblage according to the cluster for which it had the highest IndVal.

All analyses were done using the R statistical software version 2.15.2 (R Core Team, 2014). The package vegan (Oksanen et al., 2014) was used for the multivariate analysis and the MRT was built with the package mvpart (Therneau & Atkinson, 2012).

## Results

In total, 8,820 fish of 70 species were recorded in the trammel net catches, of which 7,533 fish of 62 species were included in the analyses (eight pelagic species not included). The bony fishes were represented by 49 species and the elasmobranchs by 13 species. The list of species and their frequency of occurrence is shown in Table S2. Overall, the local soft-bottom fish community was dominated by three families of benthic fish, namely Soleidae (*Solea* and *Pegusa*), Triglidae (*Chelidonichthys* and *Trigloporus*) and Bothidae (*Bothus* and *Arnoglossus*). Soleidae was the most abundant family (42%), followed by Triglidae (22%) and Bothidae (7%). In relation to biomass, Soleidae was still the dominant family (30%), although Rajidae was the second most important (28%), followed by Triglidae (12%). Details of average abundance, biomass and total length per species are shown in Tables S3 and S4.

**Figure 2:**
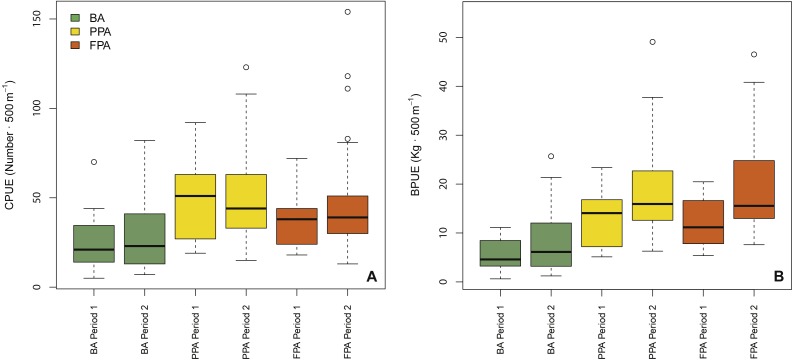
Boxplots of fish abundance and biomass. Boxplots of fish abundance (A, CPUE—n⋅500 m^−1^) and biomass (B, BPUE—kg⋅500 m^−1^) according to protection level (BA, buffer area; PPA, partially protected area; FPA, fully protected area) and period (Period 1, Before MPA Full Implementation; Period 2, After MPA Full Implementation) (Box extent, 25th to 75th percentile; Band near the middle of the box, 50th percentile/median; Whiskers range, lowest to highest datum within the 1.5 × inter-quartile range interval).

GLMM outputs (Table 1) revealed protection level and period as significant variables in the biomass model (protection level: *p* < 0.001; period: *p* = 0.03), while protection level and substrate were the significant variables in the abundance model (protection level: *p* < 0.001; substrate: *p* < 0.001). These results are supported by the boxplots shown in Fig. 2. Overall, this figure shows that abundance and biomass were higher in the PPA and FPA than in the BA, and that increases between period 1—period 2 are only apparent in biomass. The interaction of protection level and substrate was significant in both models (abundance: *p* < 0.001; biomass: *p* = 0.037), indicating a different effect of protection level according to substrate type. The coefficients indicate that mud had higher average values than sand (abundance, coef. sand = −0.15; biomass, coef. sand = −0.25), and that the sandy PPA had higher abundances in comparison to the sandy FPA (coef. FPA sand = −0.17) (Fig. S1). This pattern is different in the muddy substrate, with higher values in the FPA. Plots of abundance and biomass per protection level and substrate are shown in Fig. S1.

**Table 1:** Generalized linear mixed models of fish abundance and biomass. Results of the general linear mixed models (GLMMs) of fish abundance (CPUE n⋅500 m^−1^) and biomass (BPUE kg⋅500 m^−1^) according to protection level, period and substrate. The interaction ‘protection level × substrate’ was included and sampling campaign (*n* = 10) was considered as a random variable. GLMM of abundance and biomass used respectively, Poisson and Gamma distributions (log link).

**Response**	**Predictor**	**Fixed effect**					**Random effect**
		**Level**	**Coef.**	**St. error**	*χ*^**2**^	***p***	**St. dev.**
Abundance							
	Intercept (BA/Mud/Period 1)		3.33	0.15			
	Protection level (PL)	PPA	0.42	0.05	366.59	**<0.001**	
		FPA	0.57	0.05			
	Substrate	Sand	−0.15	0.05	28.25	**<0.001**	
	Period	Period 2	0.06	0.19	0.12	0.731	
	PL * Substrate	PPA-Sand	0.26	0.07	64.33	**<0.001**	
		FPA-Sand	−0.17	0.06			
	Sampling campaign						0.29
Biomass							
	Intercept (BA/Mud/Period 1)		1.89	0.16			
	Protection level (PL)	PPA	0.49	0.16	100.39	**<0.001**	
		FPA	0.76	0.16			
	Substrate	Sand	−0.25	0.14	0.32	0.569	
	Period	Period 2	0.33	0.15	4.71	**0.030**	
	PL * Substrate	PPA-Sand	0.50	0.20	6.61	**0.037**	
		FPA-Sand	0.13	0.19			
	Sampling campaign						0.13

**Figure 3:**
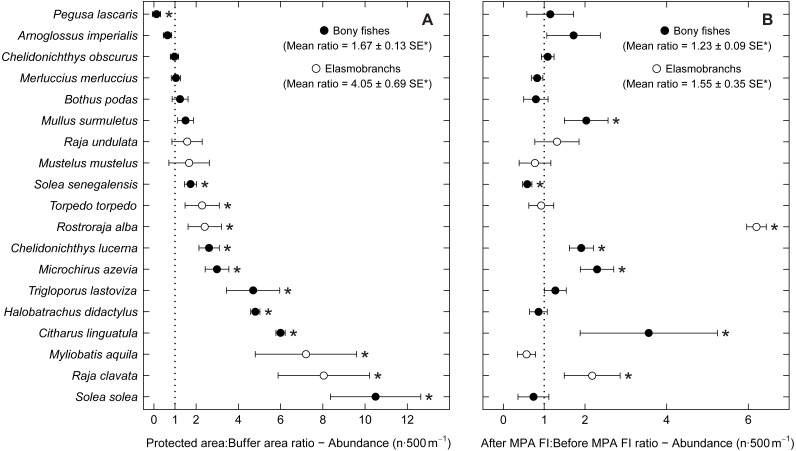
Abundance response ratio per species. Response ratios (±standard error: SE) of abundance per species for (A) Control-Impact (Protected area:Buffer area) and (B) Before-After (After MPA Full Implementation:Before MPA Full Implementation) comparisons (Protected area, fully protected area and partially protected area; Before MPA Full Implementation, Period 1; After MPA Full Implementation, Period 2; FI, Full Implementation). Protection level ratios included data from both periods and period ratios included data from the three protection levels. Ratios > 1 indicate positive responses and significant ratios (according to 95% confidence interval) are marked with ‘*’. Species ordered according to increasing Control-Impact abundance response ratio. Bony fish species are marked with ‘•’ and elasmobranch species are marked with ‘∘’.

**Figure 4:**
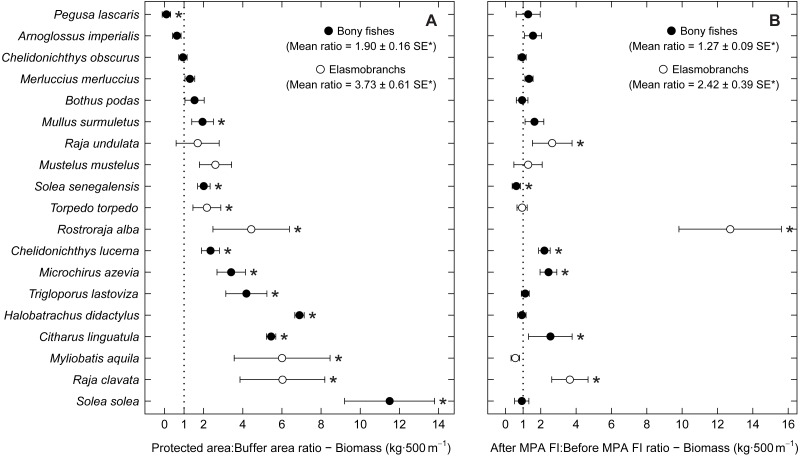
Biomass response ratio per species. Response ratios (±standard error: SE) of biomass per species for (A) Control-Impact (Protected area:Buffer area) and (B) Before-After (After MPA Full Implementation:Before MPA Full Implementation) comparisons (Protected area, fully protected area and partially protected area; Before MPA Full Implementation, Period 1; After MPA Full Implementation, Period 2; FI, Full Implementation). Protection level ratios included data from both periods and period ratios included data from the three protection levels. Ratios > 1 indicate positive responses and significant ratios (according to 95% confidence interval) are marked with ‘*’. Species ordered according to increasing Control-Impact abundance response ratio. Bony fish species are marked with ‘•’ and elasmobranch species are marked with ‘∘’.

Examining abundance and biomass ratios between the BA and higher protection levels (Figs. 3 and 4), four flatfishes (*Solea senegalensis*, *S. solea*, *C. linguatula*, *Microchirus azevia*), four elasmobranchs (*Torpedo torpedo*, *M*. *aquila, R. alba, Raja clavata*), two gurnards (*Chelidonichthys lastoviza* and *Chelidonichthys lucerna*), and the Lusitanian toadfish (*Halobatrachus didactylus*) were found to be significantly more abundant in the PPA and FPA. Of these, *R. clavata* and *S. solea* were the ones with the greatest differences. All of these species also had significantly higher biomasses in the PPA and FPA. Regarding biomass, one more species, *Mullus surmuletus,* had higher values in the PPA and FPA.

Of the eleven species with higher abundance in the higher protection levels, five also had significant increases in abundance and biomass from period 1 to period 2, namely *C. linguatula*, *R. alba*, *R. clavata*, *C. lucerna* and *M. azevia*. (Figs. 3 and 4). Other species showing positive changes over time were *M. surmuletus*, which showed an increase in abundance, and *R. undulata*, which had higher biomass in period 2. Other interesting results were the higher numbers of *Pegusa lascaris* in the BA. *Arnoglossus imperialis* showed a non-significantly higher abundance in the BA. Regarding time trends, the sole *S. senegalensis* exhibited a significant decrease in both abundance and biomass.

**Figure 5:**
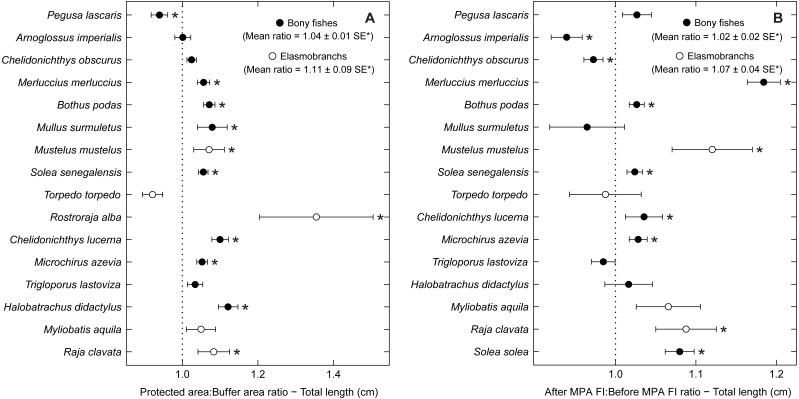
Total length response ratio per species. Response ratios (±standard error: SE) of total length per species for (A) Control-Impact (Protected area:Buffer area) and (B) Before-After (After MPA Full Implementation:Before MPA Full Implementation) comparisons (Protected area, fully protected area and partially protected area; Before MPA Full Implementation, Period 1; After MPA Full Implementation, Period 2; FI, Full Implementation). Protection level ratios included data from both periods and period ratios included data from the three protection levels. Ratios > 1 indicate positive responses and significant ratios (according to 95% confidence interval) are marked with ‘*’. Species ordered according to increasing Control-Impact abundance response ratio. Bony fish species are marked with ‘•’ and elasmobranch species are marked with ‘∘’.

Some species with positive trends in abundance and biomass also increased in average total length, including *R. alba*, *R. clavata*, *C. lucerna* and *M. azevia* (Fig. 5). Other species, with no significant trends of abundance or biomass, also showed an increase in average size, specifically *Merluccius merluccius*, *Bothus podas*, *Mustelus mustelus* and *S. solea*. *S. senegalensis*, which decreased significantly in abundance, was another species with larger average sizes in the PA and in period 2. Conversely, both *A. imperialis* and *Chelidonichthys obscurus* exhibited a decrease in average size from the first to the second period.

PERMANOVA on fish abundance and biomass (Table 2) revealed significant differences for protection level (PL) and temporal factors (period and year). The effect of protection was not demonstrated by the interactions: the terms ‘PL × period’ and ‘PL × year’ were non-significant. However, pairwise comparisons detected some differences. Specifically, the FPA became significantly different from the PPA only in period 2, and when comparing within protection level, both the PPA and FPA communities showed significant differences between periods.

**Table 2:** PERMANOVA results obtained with fish abundance and biomass. (A) PERMANOVA outputs obtained with fish abundance and biomass (Hellinger transformation), with protection level (PL, BA—buffer area; PPA, partially protected area; FPA, fully protected area), period (P, Period 1—MPA implementation period; Period 2, after MPA implementation) and year (YR) as factors (Res., Residuals). (B) Pairwise tests made per protection level within each period, and per period within each protection level.

(A) PERMANOVA								
	Abundance				Biomass			
	*df*	SS	Pseudo-F	p (perm)	*df*	SS	Pseudo-F	p (perm)
PL	2	6.51	6.5601	0.0002^***^	2	6.53	5.7795	0.0002^***^
P	1	1.6	3.0714	0.0028^**^	1	2.75	4.7146	0.0002^***^
YR	4	5.45	2.6862	0.0002^***^	4	6.68	2.9275	0.0002^***^
PL × P	2	0.92	0.9395	0.5338	2	0.95	0.8636	0.6586
PL × YR	8	3.28	0.8603	0.8340	8	3.84	0.8884	0.7920
Res. (PL + P + PL × P)	173	84.66			173	95.63		
Res. (PL + YR + PL × YR)	164	78.2			164	88.56		

(B) Pairwise tests									
		Abundance				Biomass			
		*df*	SS	Pseudo-F	p (perm)	*df*	SS	Pseudo-F	p (perm)
Period 1	BA *vs.* PPA	1	0.48	2.3526	0.0346^*^	1	0.47	2.1281	0.0424^*^
	BA *vs.* FPA	1	0.80	3.7124	0.0036^**^	1	0.83	3.6669	0.002^**^
	PPA *vs.* FPA	1	0.37	1.8301	0.0740	1	0.35	1.6347	0.1084
Period 2	BA *vs.* PPA	1	1.25	6.8167	0.0002^***^	1	1.56	7.4951	0.0002^***^
	BA *vs.* FPA	1	1.89	9.5618	0.0002^***^	1	2.13	9.7624	0.0002^***^
	PPA *vs.* FPA	1	0.46	2.9615	0.0048^**^	1	0.40	2.3556	0.0198^*^
Period 1 *vs.* Period 2	BA	1	0.35	1.5548	0.1460	1	0.37	1.4486	0.1716
	PPA	1	0.40	2.5833	0.0098^**^	1	0.53	3.0813	0.0032^**^
	FPA	1	0.33	1.8207	0.0466^*^	1	0.56	2.853	0.0094^**^

**Notes.**

* *p* < 0.05.

** *p* < 0.01.

*** *p* < 0.001.

**Figure 6:**
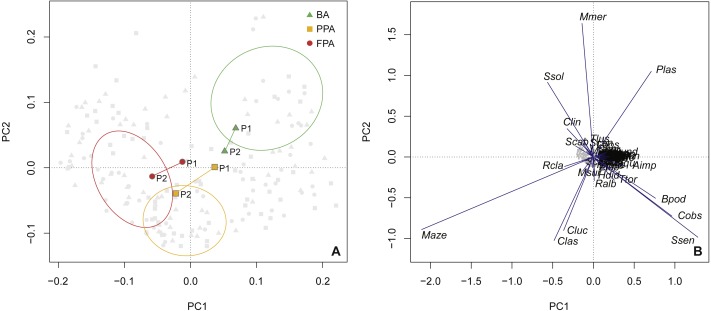
Principal components analysis of species abundance. Principal components analysis (PCA) of sites (light grey points) with transformed (Hellinger) species abundance. (A) Biplot (scaling 2) with centroids per period (period 1, period 2) and protection level (BA, buffer area; PPA, partially protected area; FPA, fully protected area), and dispersion ordiellipses (95% confidence interval) for each protection level; (B) Biplot (scaling 1) with the species vectors (see Table S2 for species codes).

The PCA ordination confirmed the results of the protection level comparison (Fig. 6A), with the BA centroids constituting a distinct group, and the PPA and FPA showing some overlap. The first axis explained 23.9% of the variation, with 34.9% of the variation explained cumulatively with the inclusion of the second axis. This analysis provides information on inter-period variation and a progression along the first axis is evident for the PPA and FPA. In both these areas, period 1 is closer to the BA group. Species vectors are shown in the second biplot (Fig. 6B). *M. azevia* (Maze), the most abundant species in our dataset, was highly related to the first component (PC1), while *M. merluccius* (Mmer) was the most correlated with the second component (PC2). Both *M. azevia* and *M. merluccius* were more abundant in the muddy substrate, and this was also the case for *S. solea* (Ssol) and *C. linguatula* (Clin). The latter two species exhibited higher abundances in the FPA, along with *R. clavata* (Rcla). *P. lascaris* (Plas), which has its vector isolated in the top-right of the plot, was more abundant in the BA and over sandy bottoms. Another species with affinity with sand was *S. senegalensis*, shown in the bottom-right. It was found in higher numbers in the PPA (Table S3—species abundance and biomass per protection level; Table S5—species abundance per substrate).

**Figure 7:**
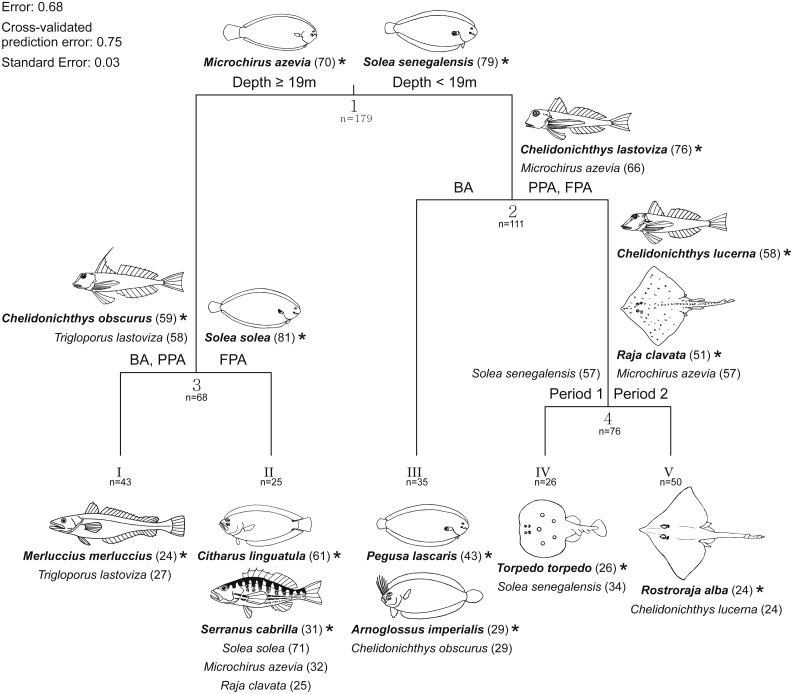
Multivariate regression tree with species abundance. Multivariate regression tree of transformed (Hellinger) fish species abundance and constrained by depth, temperature, protection level (BA, buffer area; PPA, partially protected area; FPA, fully protected area) and period (Period 1, Before MPA Full Implementation; Period 2, After MPA Full Implementation). Discriminant species (contribution to the explained variance ≥10%) with indicator values (IndVal) (Dufrêne & Legendre, 1997) ≥20 listed at each split (Arabic numbers) above the cluster to which they were allocated, and below each leaf (Roman numbers). Corresponding indicator values are reported between parentheses. Species were considered as indicator species in the cluster where they had the highest IndVal. Indicator species (bold letters) are marked with ‘*’ and illustrated (fish illustrations source: Food and Agriculture Organization of the United Nations, Original Scientific Illustrations Archive. Reproduced with permission.).

A multivariate regression tree (MRT) with four nodes and five leaves (terminal nodes) was considered the most parsimonious to represent community structure (Fig. 7). This model explained 32.2% of the species abundance variation and had an accuracy of 29% in assemblage association predictions. The primary split in the tree explained 19.6% of the species variation and separated fish assemblages according to depth, specifically depths less and greater than 19 m. These two depth intervals suggested by the primary split have a correspondence with the two sampled substrates (sand and mud). After the main distinction according to depth strata/substrate, the protection level enabled the discrimination of four assemblages, two in each substrate. In the shallow sandy area, the BA species assemblage was found to be different from the one in the PPA and FPA (7.3% of variation), while in the muddy substrate, the PPA and BA constituted a group apart from the FPA (3.1% of variation). No more nodes were created on the muddy bottom assemblages. The sandy PPA and FPA was split according to period (2.2% of variance explained).

For each split, discriminant species were identified. Significant species were allocated as indicators in the cluster for which they had the highest indicator value (IndVal) (Fig. 7). The fourteen indicator species shown in Fig. 7 were considered consistent given their high (≥50; eight species) or moderately high (≥20 and <50; six species) IndVal. Values of species mean abundance at each terminal node (leaf) are shown in Table S6. Species assigned to terminal nodes with IndVal lower than 20 (indicating some inconsistency in species allocation) are not shown in Fig. 7 and are listed in Table S7.

By taking into account the clusters with consistent indicator species (IndVal ≥ 20), several distinct assemblages can be described (Fig. 7). The main split, established by depth and substrate, separates the cluster of muddy bottom, with *M. azevia* as the most abundant species, from the sandy bottom assemblage, for which *S. senegalensis* and *C. obscurus* were assigned as indicator species. *S. solea* was considered as indicator at the FPA muddy substrate node, and the gurnards *C. lastoviza* and *C. obscurus* were discriminants of the muddy BA and PPA. The assemblage assigned for the muddy FPA terminal node included *C. linguatula*, *Serranus cabrilla* (both classified as indicators of this cluster), *S. solea*, *M. azevia* and *R. clavata*. The muddy BA and PPA community included *M. merluccius* (indicator species for this cluster) and *C. lastoviza*. With reference to the sandy stratum, two species were particularly frequent in the BA: *P. lascaris* and *A. imperialis*. *C. obscurus* was also a significant discriminant species of this group. Three other assemblages were related to sandy substrate. Firstly, *C. lastoviza* (indicator species at this cluster) and *M. azevia* appeared as discriminant species for the PPA and FPA sandy bottom assemblage. Within this cluster, *C. lucerna*, *R. clavata* (both classified as indicators of this cluster) and *M. azevia* allowed period 2 to be distinguished from period 1. *T. torpedo* and *S. senegalensis* were allocated to period 1, while *R. alba* and *C. lucerna* were assigned to period 2. It is also noteworthy that some species were discriminant for both sandy and muddy assemblages, indicating that they were not strictly associated with one habitat type. This was the case for *M. azevia*, *C. obscurus* and *C. lastoviza*.

A summary of the species ratios (abundance, biomass and size ratios according to protection and period) and MRT results is shown in Table S8. Considering the 20 species highlighted in these analyses (19 from the ratio analysis: frequency of occurrence >15%; and *S. cabrilla* from the MRT output) and their abundance and biomass time trends, three response categories can be distinguished: species with positive signs (*M. surmuletus*, *R. undulata*, *C. linguatula*, *R. alba*, *R. clavata*, *C. lucerna* and *M. azevia*), species with no detected trends (*P. lascaris*, *A. imperialis*, *C. obscurus*, *M. merluccius*, *B. podas*, *M. mustelus*, *T. torpedo*, *M. aquila*, *C. lastoviza*, *H. didactylus*, *S. cabrilla* and *S. solea*), and species with negative trends (*S. senegalensis*). To overcome the problem of habitat heterogeneity, priority was given to time trends. Additional information of life history traits (e.g., length and age at maturity, longevity), movement pattern and commercial value of these 20 species is provided in Tables S9 and S10.

## Discussion

### The soft-bottom fish community and the MPA effect

In general, our study showed spatial heterogeneity in fish assemblages. Fish abundance and biomass differed according to protection level, with higher values in the PPA and FPA in comparison with the BA. Our results also point out to the importance of substrate, which in association with depth, plays a major role structuring the communities. This is common in coastal assemblages (Claudet et al., 2006; Claudet, García-Charton & Lenfant, 2011; Pais et al., 2014). It is worth noting that not all spatial complexity was incorporated by depth, substrate and protection level, as these variables do not explain the uneven distribution of some species. One outcome of this is that in the sandy substrate, the PPA had higher abundances than the FPA, while in the muddy bottom, the FPA had higher values than the PPA. This pattern is different regarding biomass, with the sandy FPA exhibiting values similar to the PPA. *S. senegalensis*, which was more abundant in the PPA, and *R. clavata* (large species with significant contribution in biomass), commonly found in the FPA, are among the species that contributed to these trends (next section moves on to discuss species patterns). Overall, fish assemblages were mainly structured by depth and substrate, followed by protection level. With respect to protection level, some differences in abundance and biomass were found between the PPA and FPA, and the BA was noticeably a distinct group with lower values.

Examining community structure, the two soft-bottom habitats showed different patterns according to protection level. In the sandy habitat, the BA assemblage was found to be distinct from the PPA and FPA, while in the muddy substrate, the PPA and BA constituted a group apart from the FPA. Many studies have demonstrated that partial protection generally results in different communities in relation to no-take areas (Denny & Babcock, 2004; Lester & Halpern, 2008; Guidetti et al., 2014), and this was also what we observed for the muddy stratum. Habitat heterogeneity (Henriques et al., 2014) is probably related with these differences. Another possible factor is the occurrence of illegal fishing, which was reported by Cunha et al. (2011) for this area. The MPA design is another possible cause for the similarity between the muddy PPA and the BA. Because the central PPA corresponds to the area where the marine park is narrowest, about one third of this area does not include depths greater than 30 m, and the corresponding section of muddy bottom is found outside the park’s border. This discontinuity of habitat protection is expected to affect longitudinal migrations of individuals and consequently, the increase of exposure to fishing is likely to prevent effective protection benefits (Gaines et al., 2010).

Conversely, the different pattern that we found in the sandy habitat, with the PPA similar to the no-take community (also regarding abundance and biomass), might be reflecting the effectiveness of the restrictions prevailing in the PPA, meaning that the prohibitions of fishing with longlines, gill nets and trammel nets, i.e., static gear with moderate to low selectivity (Erzini et al., 2003; Erzini et al., 2006; Gonçalves et al., 2007), are effective management measures. This supports the idea that partial protection may offer effective benefits for soft-bottom fish species. This is clearly conditional on the implemented restrictions (Sciberras et al., 2013; Horta e Costa et al., 2016), and fishing gear that are generally regarded as artisanal might also have considerable impact (longlines, gillnets, trammel nets). Other factors possibly contributing to the similarity between partially protected areas and the reserve are related to geographical proximity (favoring potential movement of individuals between these areas—spillover) and site-specific effects (habitat heterogeneity; fishing pressure not equally distributed before the MPA establishment).

To surpass the complexity related to habitat differences and other site-specific effects, additional attention was given to temporal analysis. Comparisons between periods showed that although no significant abundance differences were observed, a biomass increase occurred in the PPA and FPA, with higher values after full implementation of the MPA. Fish biomass increase was the first positive effect observed in many marine reserves (García-Charton et al., 2008; Watson et al., 2009; Di Franco et al., 2012), and it was recently detected in the rocky reef fish community of this particular MPA (Horta e Costa et al., 2013). This trend is possibly related to the decrease in fishing mortality and increase in longevity, allowing fish to grow to larger sizes. Fish assemblages were also analyzed per period. Even though the interaction term of protection and time was not significant, dissimilarities between periods were found both in the PPA and FPA, suggesting that assemblages are possibly being influenced by management measures. Optimal design should have included sampling prior to the management plan implementation (Claudet & Guidetti, 2010; Donovan et al., 2016). First samples were collected with some regulations already in place and protection effect may have been larger than what our data suggests.

### The MPA effect at the species level

Overall, a wide variety of species trends and responses to spatial closure was found. Examining the three abundance and biomass response categories (positive, neutral, negative), two subgroups might be considered within the category with positive signs: species with a clear positive response (increase both in abundance and biomass) and species with only one positive indicator (increase only in abundance or biomass). The later includes *M. surmuletus*, which increased only in abundance and was not significantly more abundant in the higher protection levels, and *R. undulata*, which showed a biomass increase but no significant abundance trend. The lack of consistency in these results is likely related to the overall low abundance of these species (*R. undulata* is currently listed as endangered; IUCN, 2017), and a longer period of protection could potentially reveal more information of their trends. In this sense, they are similar to some neutral species, as some showed size increases (*M. merluccius*, *B. podas* and *M. mustelus*), others were more abundant in the PPA and FPA (*T. torpedo*, *M. aquila*, *C. lastoviza*, *H. didactylus* and *S. cabrilla*), and others were both more abundant in the higher protection levels and showed increases in size (*S. solea*).

The group with consistent positive indicators (abundance, biomass and size) includes two rays; *R. alba* and *R. clavata*; one gurnard: *C. lucerna*; and two flatfishes: *M. azevia* and *C. linguatula*. For *R. alba,* this study is the first to confirm spatial protection benefits. This is of particular importance given that this species is classified as endangered (IUCN, 2017). Spatial management seems to be a useful tool for the recovery of this species, as well as for *R. clavata*, another elasmobranch with conservation concerns, currently classified as near threatened (IUCN, 2017). Positive responses to spatial management were previously reported for *R. clavata* in the Mediterranean by Dimech et al. (2008). Hunter et al. (2006) and Wiegand, Hunter & Dulvy (2011) also concluded that spatial closures were an appropriate approach for this species in the North Sea. Although the distribution of the local populations is probably wider than the MPA, many individuals might be using the area on a regular basis, given the known tendency to repeatedly return to specific locations (Hunter et al., 2005; Ellis et al., 2011).

Concerning *C. lucerna*, few studies have addressed its abundance in protected areas, despite its commercial importance. Piet & Rijnsdorp (1998) reported an increase in abundance of this species after the establishment of the “plaice box” in the North Sea, while Guidetti & Claudet (2010) found that it occurred in lower abundances inside the Torre Guaceto MPA (Italy) in comparison to the outside area. Our results show that this species can benefit from spatial closures. Within the Soleidae species (represented by nine species, four of which with frequency of occurrence >15%), *M. azevia* was the one presenting more positive indicators. It showed increases in abundance, biomass and size, and these trends may be related to the implemented regulations. As for the flounder *C. linguatula*, no trends over time were found in the study by Iannibelli & Musmarra (2008) in an area with trawling restrictions, while in our results, this was the only non-target species with consistent positive indicators. Claudet et al. (2010) pointed out that bycatch species may also be affected by fishing pressure or habitat degradation, which seems to be the case for this species.

The only species that exhibited a decrease in abundance was *S. senegalensis*, even though it was more abundant in the protected area and showed a size increase. This abundance decrease might be related with migrations to nearby estuaries (Andrade, 1990; Vinagre, Costa & Cabral, 2007) or to fishing grounds outside the reserve, with increasing exposure and vulnerability to gillnets and trammel nets. This phenomenon was hypothesized by Abecasis, Afonso & Erzini (2014) for this species in this same MPA (acoustic telemetry data).

It is also noteworthy that some species showed affinity to the BA, particularly *P. lascaris* and *A imperialis*. Both species had low frequency of occurrence and they exhibited restricted distributions in the study area. Similarly, *C. linguatula* was also unevenly distributed, but it showed affinity with the muddy bottoms in the FPA. This is in accordance with previous studies, that identified species with pronounced site-specific responses within MPAs (Claudet, García-Charton & Lenfant, 2011; Eddy, Pande & Gardner, 2014). One possibility is that their patchy distributions are related to biotic factors such as niche breadth, perhaps habitat preferences and/or prey availability. Studies such as the conducted by Ross (1986) have shown that among fish assemblages, the availability of food items is commonly an important factor for habitat discrimination. Moreover, it is argued that specialist species are more vulnerable to environmental stressors and exploitation, and that their dependence on habitat heterogeneity is also higher (Wilson et al., 2008; Slatyer, Hirst & Sexton, 2013).

As with rocky reef fish assemblages (Micheli et al., 2004; Ashworth & Ormond, 2005; Blyth-Skyrme et al., 2006), soft-bottom fishes exhibit a wide diversity of responses to spatial regulations. Our results suggest that part of this variability is connected with habitat heterogeneity. Still, the cause of site-specific preferences of some of the studied species is not fully understood. Studies of species movement ecology would provide insights for both habitat preferences and migratory behavior. Our data illustrates this gap of knowledge, as some mismatches related with migratory species were found. For instance, both *R. clavata* and *R. alba* demonstrate that species with yearly migrations can benefit from spatial measures, in accordance with the results of other studies (e.g., Claudet et al., 2010). However, it would be simplistic to infer that home range does not play a role in the effectiveness of spatial protection. Instead, possible connections are probably more complex, as illustrated by the species *M. mustelus*, a species that shares life history traits with the rays. This species did not exhibit consistent positive response to protection, and its migratory habits might contribute for this (strictly migrant *vs* seasonally migrant). The complexity of the effect of movement patterns in species response to protection was pointed out in previous studies (Claudet et al., 2010; Villegas-Ríos, Moland & Olsen, 2017).

In relation to species traits and their role in the response to protection, our most compelling results point out to body size as a possible factor. In our study, this is supported by the two rays (*R. clavata* and *R. alba*), two elasmobranchs with large body size and late maturity. This is in accordance with the observations of other authors (Claudet et al., 2006; García-Charton et al., 2008; Claudet et al., 2010; Sciberras et al., 2015), who reported body size as an important trait related to spatial protection. This might be due to the fact that larger fish may be more easily caught by fishing gear, and also because large body size generally reflects specific life history traits such as late maturity (Hutchings et al., 2012).

### Implications and suggestions for future research

In this study, there was some evidence of protection effectiveness, supported by the significant increase in biomass in the protected areas. However, like in rocky habitats, effectiveness is cross-linked with habitat and species characteristics. In relation to habitat, it is important to identify habitat requirements of species that are protection targets, and habitat mapping should be carried out before the management plan development. This was also emphasized by other authors for rocky bottoms (e.g., Gaines et al., 2010; Halpern, Lester & McLeod, 2010). In addition, we recommend that habitat continuity should be taken into account in the design of MPAs aiming to protect soft-bottom fishes. Furthermore, we highlight the importance of enforcement measures, as compliance is essential for effective protection, long-term benefits and appropriate monitoring (e.g., Bergseth, Russ & Cinner, 2015). With respect to species characteristics, we highlight body size as a possible factor of response to protection. The inclusion of longer time scales would provide information on this and other underlying factors, and also reveal if more species could respond positively to spatial regulations. Future research on fish movements (telemetry), habitat requirements and trophic ecology (stable isotopes) would also offer useful knowledge to better understand how spatial protection measures should be used in the management and conservation of soft-bottom fish species.

##  Supplemental Information

10.7717/peerj.4653/supp-1 Figure S1 Barplots of fish abundance and biomass per protection level and substrate Barplots of mean (±standard error) fish abundance (A: CPUE n⋅500 m^−1^) and biomass (B: BPUE kg⋅500 m^−1^) per protection level and substrate (sand, mud).

10.7717/peerj.4653/supp-2 Table S1 List of fish species sampled during the experimental fishing surveys List of fish species sampled during the experimental fishing surveys in the Luiz Saldanha Marine Park, and corresponding references (full references in File S1) used for length-weight (L-W) relationships (for all species except pelagics). The environment, maximum length reported (Froese and Pauly 2017, fishbase.org) and fisheries category (target, bycatch, non-commercial) of each species are also shown. (1) Dimension considered: disc width.

10.7717/peerj.4653/supp-3 Table S2 Species frequency of occurrence Table with frequency of occurrence of the species included in the analyses (FO%; percentage of samples with presence of each species). Species code (used in Fig. 6) and family are also shown (species ordered according to decreasing FO%).

10.7717/peerj.4653/supp-4 Table S3 Mean species abundance and biomass for each protection level and period Mean (and standard error - SE) species abundance (CPUE - n⋅500 m^−1^) and biomass (BPUE - kg⋅500 m^−1^) according to period (Period 1, Period 2) and protection level (BA - buffer area; PPA - partially protected area; FPA - fully protected area). Only species with frequency of occurrence higher than 15% are shown.

10.7717/peerj.4653/supp-5 Table S4 Mean species total length for each protection level and period Mean total length (TL - cm) per species according to period (Period 1, Period 2) and protection level (BA - buffer area; PPA - partially protected area; FPA - fully protected area). Only species with frequency of occurrence higher than 15% are shown.

10.7717/peerj.4653/supp-6 Table S5 Mean species abundance for each substrate type Mean (and standard error - SE) species abundance (CPUE - n⋅500 m^−1^) per substrate type. Only species with frequency of occurrence higher than 15% are shown.

10.7717/peerj.4653/supp-7 Table S6 Mean species abundance in each leaf - multivariate regression tree Mean (and standard error - SE) species abundance (CPUE - n⋅500 m^−1^) in each leaf of the multivariate regression tree analysis (MRT) (Leaf I - Muddy BA & PPA; Leaf II - Muddy FPA; Leaf III - Sandy BA; Leaf IV - Sandy PPA & FPA, Period 1; Leaf V - Sandy PPA & FPA, Period 2).

10.7717/peerj.4653/supp-8 Table S7 Discriminant species at each leaf of the multivariate regression tree Discriminant species at each leaf (terminal node) of the multivariate regression tree analysis (Leaf I - Muddy BA & PPA; Leaf II - Muddy FPA; Leaf III - Sandy BA; Leaf IV - Sandy PPA & FPA, Period 1; Leaf V - Sandy PPA & FPA, Period 2). Indicator values (IndVal) are shown (species ordered according to decreasing IndVal) and the symbol ‘*’ signalizes the indicator species (highest IndVal obtained for that species was in that node) (note: discriminant species with IndVal ≥ 20 are shown in Fig. 7).

10.7717/peerj.4653/supp-9 Table S8 Summary table of the ratios analyses and multivariate regression tree Summary table of the ratio (Protected area:Buffer area; After MPA Full Implementation:Before MPA Full Implementation) analyses (N - abundance, B - biomass, L - total length) and multivariate regression tree (MRT) (MRT Ind Spp - cluster where the species was assigned as indicator) (BA - buffer area; PPA - partially protected area; FPA - fully protected area; PA - protected area: PPA & FPA; Period 1 - Before MPA Full Implementation; Period 2 - After MPA Full Implementation) (↑ - Increase; ↓ - Decrease). Species included in the ratios analyses and obtained in the MRT output with IndVal ≥ 20 are shown. (1) Species not included in the ratios analyses; (2) Species with less than 10 individuals in the BA; (3) Species with less than 10 individuals in Period 1; (4) Species with indicator value (IndVal) < 20.

10.7717/peerj.4653/supp-10 Table S9 Table of additional information of species traits: fisheries category and reproduction Additional information of species traits: taxonomic family, environment, maximum length, market price and reproduction (mode, length and age at maturity) (references of length/age at maturity listed). Fisheries category is shown in the ‘price per kg’ column: T - target, B - bycatch, NC - non-commercial. Species ranked according to abundance (N) and biomass (B) time trend category (2nd column; see Table S8). (1) Information not found; (2) Disc width; (3) Hermaphroditic.

10.7717/peerj.4653/supp-11 Table S10 Table of additional information of species traits: longevity and movement pattern Additional information of species traits: maximum reported age, theoretical longevity (based on Von Bertalanffy Growth Function - VBGF) and movement pattern. Information on the type of study reporting movement pattern data is shown: FS - fishing surveys, MR - mark-recapture, OC - otolith chemistry, SI - stable isotopes, T - telemetry, UVC - underwater visual census, NS - not specified. Species ranked according to abundance (N) and biomass (B) time trend category (2nd column; see Table S8). (1) Information not found; (2) Based on congener species.

10.7717/peerj.4653/supp-12 File S1 References used in the Supplemental Information section

10.7717/peerj.4653/supp-13 Supplemental Information 1 Dataset of experimental fishing surveys Raw data of experimental fishing surveys in the Luiz Saldanha Marine Park.

## Additional Information and Declarations

### Field Study Permissions

The following information was supplied relating to field study approvals (i.e., approving body and any reference numbers): The Portuguese Institute for Nature Conservation and Forests (Instituto de Conservação da Natureza e Florestas—ICNF) approved the field surveys for this study.

### Data Availability

The following information was supplied regarding data availability: The raw data is supplied as a Supplemental File.

## References

[ref-1] Abecasis D, Afonso P, Erzini K (2014). Can small MPAs protect local populations of a coastal flatfish, *Solea senegalensis*?. Fisheries Management and Ecology.

[ref-2] Anderson MJ (2001). A new method for non-parametric multivariate analysis of variance. Austral Ecology.

[ref-3] Andrade JP (1990). A importância da Ria Formosa no ciclo biológico de *Solea senegalensis* (Kaup 1858), *Solea vulgaris* (Quensel 1806), *Solea lascaris* (Risso 1810) e *Microchirus azevia* (Capello, 1868). PhD Thesis.

[ref-4] Ashworth JS, Ormond RFG (2005). Effects of fishing pressure and trophic group on abundance and spillover across boundaries of a no-take zone. Biological Conservation.

[ref-5] Audzijonyte A, Kuparinen A (2016). The role of life histories and trophic interactions in population recovery: life histories and population recovery. Conservation Biology.

[ref-6] Bergseth BJ, Russ GR, Cinner JE (2015). Measuring and monitoring compliance in no-take marine reserves. Fish and Fisheries.

[ref-7] Blyth-Skyrme RE, Kaiser MJ, Hiddink JG, Edwards-Jones G, Hart PJB (2006). Conservation benefits of temperate marine protected areas: variation among fish species. Conservation Biology.

[ref-8] Breslow NE, Clayton DG (1993). Approximate inference in generalized linear mixed models. Journal of the American Statistical Association.

[ref-9] Bruce T, Meirelles PM, Garcia G, Paranhos R, Rezende CE, De Moura RL, Filho R-F, Coni EOC, Vasconcelos AT, Amado Filho G, Hatay M, Schmieder R, Edwards R, Dinsdale E, Thompson FL (2012). Abrolhos Bank reef health evaluated by means of water quality, microbial diversity, benthic cover, and fish biomass data. PLOS ONE.

[ref-10] Byers JE, Noonburg EG (2007). Poaching, enforcement, and the efficacy of marine reserves. Ecological Applications.

[ref-11] Campbell SJ, Hoey AS, Maynard J, Kartawijaya T, Cinner J, Graham NAJ, Baird AH (2012). Weak compliance undermines the success of no-take zones in a large government-controlled marine protected area. PLOS ONE.

[ref-12] Chapman DD, Pikitch EK, Babcock E, Shivji MS (2005). Marine reserve design and evaluation using automated acoustic telemetry: a case-study involving coral reef-associated sharks in the Mesoamerican Caribbean. Marine Technology Society Journal.

[ref-13] Claudet J, García-Charton JA, Lenfant P (2011). Combined effects of levels of protection and environmental variables at different spatial resolutions on fish assemblages in a marine protected area: species-habitat relations and MPAs. Conservation Biology.

[ref-14] Claudet J, Guidetti P (2010). Improving assessments of marine protected areas. Aquatic Conservation: Marine and Freshwater Ecosystems.

[ref-15] Claudet J, Osenberg CW, Benedetti-Cecchi L, Domenici P, García-Charton J-A, Pérez-Ruzafa Á, Badalamenti F, Bayle-Sempere J, Brito A, Bulleri F, Culioli J-M, Dimech M, Falcón JM, Guala I, Milazzo M, Sánchez-Meca J, Somerfield PJ, Stobart B, Vandeperre F, Valle C, Planes S (2008). Marine reserves: size and age do matter. Ecology Letters.

[ref-16] Claudet J, Osenberg CW, Domenici P, Badalamenti F, Milazzo M, Falcón JM, Bertocci I, Benedetti-Cecchi L, García-Charton JA, Goñi R, Borg JA, Forcada A, De Lucia GA, Pérez-Ruzafa Á, Afonso P, Brito A, Guala I, Le Diréach L, Sanchez-Jerez P, Somerfield PJ, Planes S (2010). Marine reserves: fish life history and ecological traits matter. Ecological Applications.

[ref-17] Claudet J, Pelletier D, Jouvenel J-Y, Bachet F, Galzin R (2006). Assessing the effects of marine protected area (MPA) on a reef fish assemblage in a northwestern Mediterranean marine reserve: Identifying community-based indicators. Biological Conservation.

[ref-18] Cunha AH, Erzini K, Serrão EA, Gonçalves EJ, Borges R, Henriques M, Henriques V, Guerra M, Marbá N, Fonseca M (2011). LIFE-Biomares—Restoration and management of biodiversity in the marine park site Arrábida-Espichel (LIFE06 NAT/P/000192). Final report.

[ref-19] De’Ath G (2002). Multivariate regression trees: a new technique for modeling species-environment relationships. Ecology.

[ref-20] Denney NH, Jennings S, Reynolds JD (2002). Life–history correlates of maximum population growth rates in marine fishes. Proceedings of the Royal Society of London B: Biological Sciences.

[ref-21] Denny CM, Babcock RC (2004). Do partial marine reserves protect reef fish assemblages?. Biological Conservation.

[ref-22] Di Franco A, Gillanders BM, De Benedetto G, Pennetta A, De Leo GA, Guidetti P (2012). Dispersal patterns of coastal fish: implications for designing networks of marine protected areas. PLOS ONE.

[ref-23] Dimech M, Camilleri M, Hiddink JG, Kaiser MJ, Ragonese S, Schembri PJ (2008). Differences in demersal community structure and biomass size spectra within and outside the Maltese Fishery Management Zone (FMZ). Scientia Marina.

[ref-24] Donovan MK, Friedlander AM, Usseglio P, Goodell W, Iglesias I, Schemmel EM, Stamoulis KA, Filous A, Giddens J, Kamikawa K, Koike H, McCoy K, Wall CB (2016). Effects of gear restriction on the abundance of juvenile fishes along sandy beaches in Hawai‘i. PLOS ONE.

[ref-25] Dufrêne M, Legendre P (1997). Species assemblages and indicator species: the need for a flexible asymmetrical approach. Ecological Monographs.

[ref-26] Eddy TD, Pande A, Gardner JPA (2014). Massive differential site-specific and species-specific responses of temperate reef fishes to marine reserve protection. Global Ecology and Conservation.

[ref-27] Ellis JR, Morel G, Burt G, Bossy S (2011). Preliminary observations on the life history and movements of skates (Rajidae) around the Island of Jersey, western English Channel. Journal of the Marine Biological Association of the United Kingdom.

[ref-28] Erzini K, Gonçalves JMS, Bentes L, Lino PG, Ribeiro J, Stergiou KI (2003). Quantifying the roles of competing static gears: comparative selectivity of longlines and monofilament gill nets in a multi-species fishery of the Algarve (southern Portugal). Scientia Marina.

[ref-29] Erzini K, Gonçalves JMS, Bentes L, Moutopoulos DK, Casal JAH, Soriguer MC, Puente E, Errazkin LA, Stergiou KI (2006). Size selectivity of trammel nets in southern European small-scale fisheries. Fisheries Research.

[ref-30] Fisher JAD, Frank KT (2002). Changes in finfish community structure associated with an offshore fishery closed area on the Scotian Shelf. Marine Ecology Progress Series.

[ref-31] Fréon P, Misund OA (1999). Dynamics of pelagic fish distribution and behaviour: Effects on fisheries and stock assessment.

[ref-32] Gaines SD, White C, Carr MH, Palumbi SR (2010). Designing marine reserve networks for both conservation and fisheries management. Proceedings of the National Academy of Sciences of the United States of America.

[ref-33] García-Charton JA, Pérez-Ruzafa Á (1999). Ecological heterogeneity and the evaluation of the effects of marine reserves. Fisheries Research.

[ref-34] García-Charton JA, Pérez-Ruzafa A, Marcos C, Claudet J, Badalamenti F, Benedetti-Cecchi L, Falcón JM, Milazzo M, Schembri PJ, Stobart B, Vandeperre F, Brito A, Chemello R, Dimech M, Domenici P, Guala I, Le Diréach L, Maggi E, Planes S (2008). Effectiveness of European Atlanto-Mediterranean MPAs: do they accomplish the expected effects on populations, communities and ecosystems?. Journal for Nature Conservation.

[ref-35] Gell FR, Roberts CM (2003). Benefits beyond boundaries: the fishery effects of marine reserves. Trends in Ecology & Evolution.

[ref-36] Giakoumi S, Kokkoris GD (2013). Effects of habitat and substrate complexity on shallow sublittoral fish assemblages in the Cyclades Archipelago, North-eastern Mediterranean Sea. Mediterranean Marine Science.

[ref-37] Gonçalves EJ, Henriques M, Almada VC, Beumer JP, Grant A, Smith DC (2002). Use of temperate reef fish community to identify priorities in the establishment of a marine protected area. Aquatic protected areas: what works best and how do we know? Proceedings of the World Congress on Aquatic Protected Areas.

[ref-38] Gonçalves JMS, Stergiou KI, Hernando JA, Puente E, Moutopoulos DK, Arregi L, Soriguer MC, Vilas C, Coelho R, Erzini K (2007). Discards from experimental trammel nets in southern European small-scale fisheries. Fisheries Research.

[ref-39] Grüss A, Kaplan DM, Guénette S, Roberts CM, Botsford LW (2011). Consequences of adult and juvenile movement for marine protected areas. Biological Conservation.

[ref-40] Guidetti P, Baiata P, Ballesteros E, Di Franco A, Hereu B, Macpherson E, Micheli F, Pais A, Panzalis P, Rosenberg AA, Zabala M, Sala E (2014). Large-scale assessment of Mediterranean marine protected areas effects on fish assemblages. PLOS ONE.

[ref-41] Guidetti P, Claudet J (2010). Comanagement practices enhance fisheries in marine protected areas. Conservation Biology.

[ref-42] Halpern BS, Lester SE, McLeod KL (2010). Placing marine protected areas onto the ecosystem-based management seascape. Proceedings of the National Academy of Sciences of the United States of America.

[ref-43] Halpern BS, Warner RR (2002). Marine reserves have rapid and lasting effects. Ecology Letters.

[ref-44] Henriques M, Gonçalves E, Almada VC, Beumer JP, Grant A, Smith DC (1999). The conservation of littoral fish communities: a case study at Arrábida coast (Portugal). Behaviour and conservation of littoral fishes.

[ref-45] Henriques V, Guerra MT, Mendes B, Gaudêncio MJ, Fonseca P (2014). Benthic habitat mapping in a Portuguese Marine Protected Area using EUNIS: an integrated approach. Journal of Sea Research.

[ref-46] Horta e Costa B, Claudet J, Franco G, Erzini K, Caro A, Gonçalves EJ (2016). A regulation-based classification system for Marine Protected Areas (MPAs). Marine Policy.

[ref-47] Horta e Costa B, Erzini K, Caselle J, Folhas H, Gonçalves E (2013). “Reserve effect” within a temperate marine protected area in the north-eastern Atlantic (Arrábida Marine Park, Portugal). Marine Ecology Progress Series.

[ref-48] Hunter E, Berry F, Buckley AA, Stewart C, Metcalfe JD (2006). Seasonal migration of thornback rays and implications for closure management: ray migration and closure management. Journal of Applied Ecology.

[ref-49] Hunter E, Buckley AA, Stewart C, Metcalfe JD (2005). Migratory behaviour of the thornback ray, *Raja clavata*, in the southern North Sea. Journal of the Marine Biological Association of the United Kingdom.

[ref-50] Hutchings JA, Myers RA, García VB, Lucifora LO, Kuparinen A (2012). Life-history correlates of extinction risk and recovery potential. Ecological Applications.

[ref-51] Iannibelli M, Musmarra D (2008). Effects of anti-trawling artificial reefs on fish assemblages: the case of Salerno Bay (Mediterranean Sea). Italian Journal of Zoology.

[ref-52] IUCN (2017). http://www.iucnred.

[ref-53] Jennings S, Greenstreet SPR, Reynolds JD (1999). Structural change in an exploited fish community: a consequence of differential fishing effects on species with contrasting life histories. Journal of Animal Ecology.

[ref-54] Legendre P, Gallagher E (2001). Ecologically meaningful transformations for ordination of species data. Oecologia.

[ref-55] Lester S, Halpern B (2008). Biological responses in marine no-take reserves versus partially protected areas. Marine Ecology Progress Series.

[ref-56] Lima FP, Ribeiro PA, Queiroz N, Hawkins SJ, Santos AM (2007). Do distributional shifts of northern and southern species of algae match the warming pattern?. Global Change Biology.

[ref-57] Lubchenco J, Palumbi SR, Gaines SD, Andelman S (2003). Plugging a hole in the ocean: the emerging science of marine reserves 1. Ecological Applications.

[ref-58] Micheli F, Halpern BS, Botsford LW, Warner RR (2004). Trajectories and correlates of community change in no-take marine reserves. Ecological Applications.

[ref-59] Murawski SA, Brown R, Lai H-L, Rago PJ, Hendrickson L (2000). Large-scale closed areas as a fishery-management tool in temperate marine systems: the Georges Bank experience. Bulletin of Marine Science.

[ref-60] Myers RA, Worm B (2003). Rapid worldwide depletion of predatory fish communities. Nature.

[ref-61] Oksanen J, Blanchet FG, Kindt R, Legendre P, Minchin PR, O’Hara RB, Simpson GL, Solymos P, Stevens MHH, Wagner H (2014). http://cran.r-project.org/web/packages/vegan.

[ref-62] Osenberg CW, Shima JS, Miller SL, Stier AC, Claudet J (2011). Assessing effects of marine protected areas: confounding in space and possible solutions. Marine protected areas—effects, networks and monitoring—a multidisciplinary approach.

[ref-63] Pais MP, Henriques S, Murta AG, Azevedo M, Costa MJ, Cabral HN (2014). Identifying functional homogeneity in a dynamic environment: application to soft-substrate fish assemblages off the Portuguese coast. Journal of Sea Research.

[ref-64] Pauly D, Palomares M-L (2005). Fishing down marine food web: it is far more pervasive than we thought. Bulletin of Marine Science.

[ref-65] Payton ME, Greenstone MH, Schenker N (2003). Overlapping confidence intervals or standard error intervals: what do they mean in terms of statistical significance?. Journal of Insect Science.

[ref-66] Piet GJ, Rijnsdorp AD (1998). Changes in the demersal fish assemblage in the south-eastern North Sea following the establishment of a protected area (“plaice box”). ICES Journal of Marine Science: Journal Du Conseil.

[ref-67] Pipitone C, Badalamenti F, D’Anna G, Patti B (2000). Fish biomass increase after a four-year trawl ban in the Gulf of Castellammare (NW Sicily, Mediterranean Sea). Fisheries Research.

[ref-68] R Core Team (2014). www.R-project.org.

[ref-69] Roberts CM (1997). Ecological advice for the global fisheries crisis. Trends in Ecology and Evolution.

[ref-70] Roberts CM, Polunin NVC (1993). Marine reserves: simple solutions to sanaging somplex fisheries?. Ambio.

[ref-71] Ross ST (1986). Resource partitioning in fish assemblages: a review of field studies. Copeia.

[ref-72] Russ GR, Alcala AC (2004). Marine reserves: long-term protection is required for full recovery of predatory fish populations. Oecologia.

[ref-73] Sala E, Ballesteros E, Dendrinos P, Di Franco A, Ferretti F, Foley D, Fraschetti S, Friedlander A, Garrabou J, Güçlüsoy H, Guidetti P, Halpern BS, Hereu B, Karamanlidis AA, Kizilkaya Z, Macpherson E, Mangialajo L, Mariani S, Micheli F, Pais A, Riser K, Rosenberg AA, Sales M, Selkoe KA, Starr R, Tomas F, Zabala M (2012). The structure of mediterranean rocky reef ecosystems across environmental and human gradients, and conservation implications. PLOS ONE.

[ref-74] Sciberras M, Jenkins SR, Kaiser MJ, Hawkins SJ, Pullin AS (2013). Evaluating the biological effectiveness of fully and partially protected marine areas. Environmental Evidence.

[ref-75] Sciberras M, Jenkins SR, Mant R, Kaiser MJ, Hawkins SJ, Pullin AS (2015). Evaluating the relative conservation value of fully and partially protected marine areas. Fish and Fisheries.

[ref-76] Silva C, Kerwath S, Attwood C, Thorstad E, Cowley P, Økland F, Wilke C, Næsje T (2013). Quantifying the degree of protection afforded by a no-take marine reserve on an exploited shark. African Journal of Marine Science.

[ref-77] Slatyer RA, Hirst M, Sexton JP (2013). Niche breadth predicts geographical range size: a general ecological pattern. Ecology Letters.

[ref-78] Tetreault I, Ambrose RF (2007). Temperate marine reserves enhance targeted but not untargeted fishes in multiple no-take MPAs. Ecological Applications.

[ref-79] Therneau TM, Atkinson B (2012). http://cran.r-project.org/web/packages/rpart.

[ref-80] Underwood AJ (1992). Beyond BACI: the detection of environmental impacts on populations in the real, but variable, world. Journal of Experimental Marine Biology and Ecology.

[ref-81] Underwood AJ (1994). On Beyond BACI: sampling designs that might reliably detect environmental disturbances. Ecological Applications.

[ref-82] Vasconcelos L, Ramos Pereira MJ, Caser U, Gonçalves G, Silva F, Sá R (2013). MARGov—setting the ground for the governance of marine protected areas. Ocean & Coastal Management.

[ref-83] Villegas-Ríos D, Moland E, Olsen EM (2017). Potential of contemporary evolution to erode fishery benefits from marine reserves. Fish and Fisheries.

[ref-84] Vinagre C, Costa MJ, Cabral HN (2007). Impact of climate and hydrodynamics on sole larval immigration towards the Tagus estuary, Portugal. Estuarine, Coastal and Shelf Science.

[ref-85] Vincent AC, Hall HJ (1996). The threatened status of marine fishes. Trends in Ecology & Evolution.

[ref-86] Watson D, Anderson M, Kendrick G, Nardi K, Harvey E (2009). Effects of protection from fishing on the lengths of targeted and non-targeted fish species at the Houtman Abrolhos Islands, Western Australia. Marine Ecology Progress Series.

[ref-87] Wiegand J, Hunter E, Dulvy NK (2011). Are spatial closures better than size limits for halting the decline of the North Sea thornback ray, *Raja clavata*?. Marine and Freshwater Research.

[ref-88] Wilson SK, Burgess SC, Cheal AJ, Emslie M, Fisher R, Miller I, Polunin NVC, Sweatman HPA (2008). Habitat utilization by coral reef fish: implications for specialists vs. generalists in a changing environment. Journal of Animal Ecology.

[ref-89] Zuur AF, Ieno EN, Walker NJ, Saveliev AA, Smith GM (2009). Mixed effects models and extensions in ecology with R.

