# Supplementary figures and images for: Soft-bottom fishes and spatial protection: findings from a temperate marine protected area
Source: PeerJ. 2018 Jun 8;6:e4653. doi: 10.7717/peerj.4653 (PMC5995104; doi:10.7717/peerj.4653)

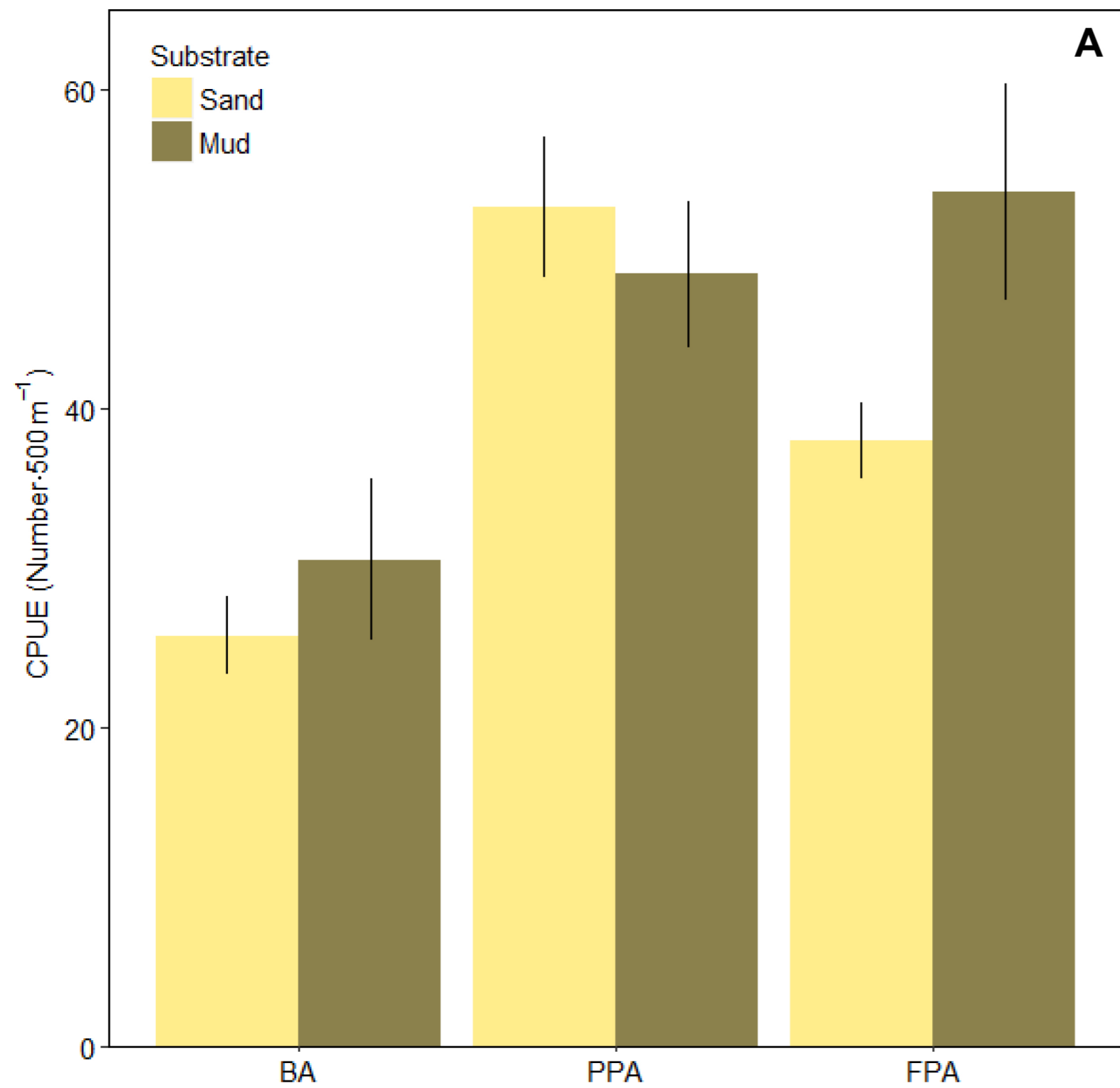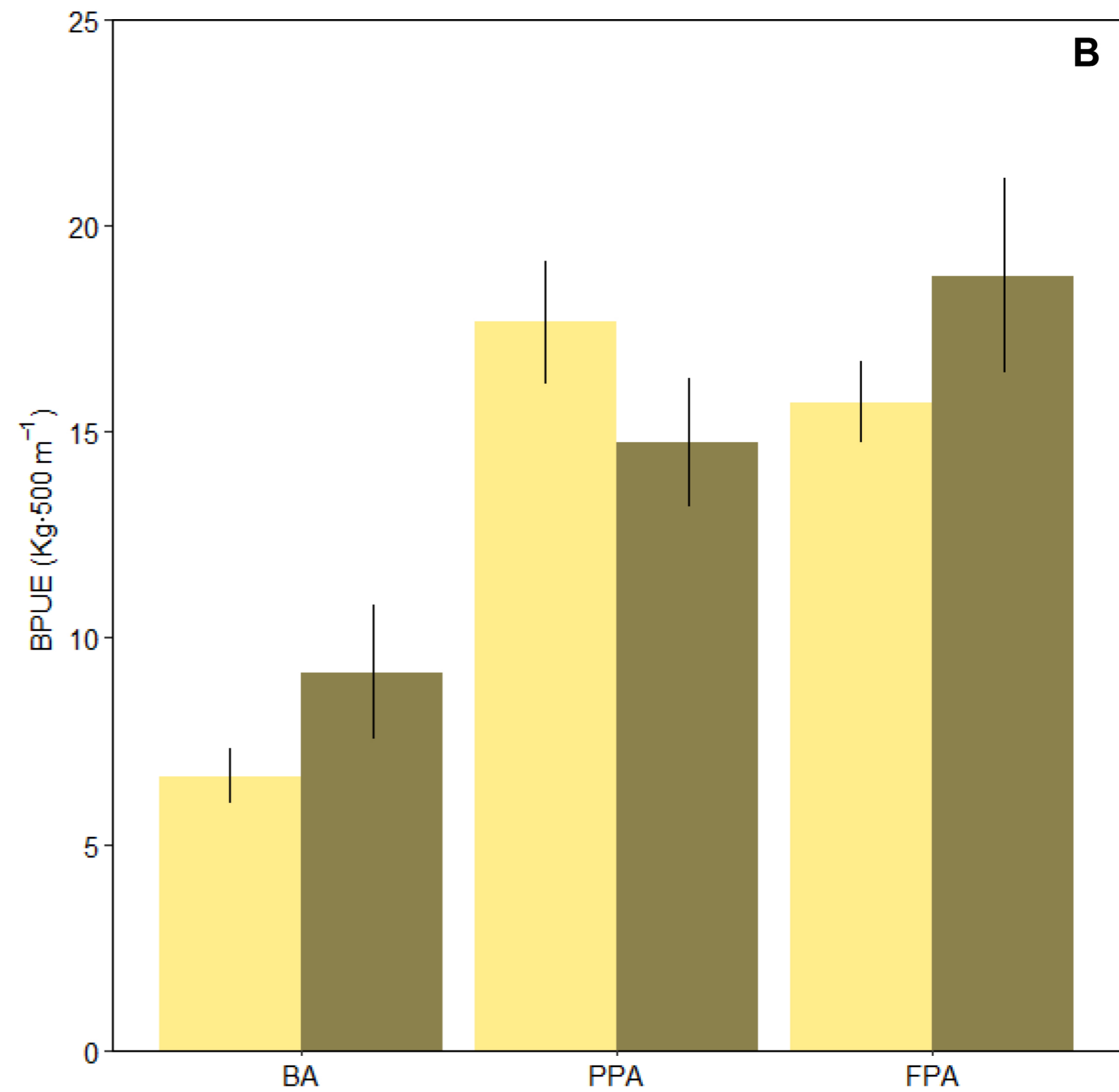

Supplement: Figure S1 — Barplots of mean (±standard error) fish abundance (A: CPUE n⋅500 m−1) and biomass (B: BPUE kg⋅500 m−1) per protection level and substrate (sand, mud). [file peerj-06-4653-s001.pdf]
