# Supplementary material for: Soft-bottom fishes and spatial protection: findings from a temperate marine protected area
Source: PeerJ. 2018 Jun 8;6:e4653. doi: 10.7717/peerj.4653 (PMC5995104; doi:10.7717/peerj.4653)
Supplement: File S1 [file peerj-06-4653-s012.pdf]

REFERENCES (Supp. Info.)

Abecasis D., Afonso P., Erzini K. 2014. Can small MPAs protect local populations of a coastal flatfish, *Solea senegalensis* ? *Fisheries Management and Ecology* 21:175–185. DOI: 10.1111/fme.12061.

Afonso-Dias I., Reis C., Andrade JP. 2005. Reproductive aspects of *Microchirus azevia* (Risso, 1810) (Pisces: Soleidae) from the south coast of Portugal. *Scientia Marina* 69:275–283.

Akalin S., Ilhan D. 2013. Age, Growth and Mortality of Rock Gurnard (*Trigloporus lastoviza* (Bonnaterre, 1788)) (Osteichthyes:Triglidae) in İzmir Bay. *The Black Sea Journal of Sciences* 3:47–56.

Alós J., March D., Palmer M., Grau A., Morales-Nin B. 2011. Spatial and temporal patterns in *Serranus cabrilla* habitat use in the NW Mediterranean revealed by acoustic telemetry. *Marine Ecology Progress Series* 427:173–186.

Andrade JP. 1990. A importância da Ria Formosa no ciclo biológico de *Solea senegalensis* (Kaup 1858), *Solea vulgaris* (Quensel 1806), *Solea lascaris* (Risso 1810) e *Microchirus azevia* (Capello, 1868). D. Phil. Thesis, Universidade do Algarve.

Andrade JP. 1998. Age and growth of the bastard sole, *Microchirus azevia* (Capello, 1868) (Pisces, Soleidae) from the south coast of Portugal. *Fisheries Resources* 34:205–208.

Arslan M., Ismen A. 2013. Age, growth and reproduction of *Mullus surmuletus* (Linnaeus, 1758) in Saros Bay (Northern Aegean Sea). *Journal of the Black Sea / Mediterranean Environment* 19:217–233.

Baron J. 1985. Les triglides (Téléostéens, Scorpaeniformes) de la baie de Douarnenez. II. La reproduction de: *Eutrigla gurnardus, Trigla lucerna, Trigloporus lastoviza* et *Aspitrigla cuculus*. *Cybium* 9:255–281.

Barreiros JP., Rodeia J. 2004. Preliminary results of reproductive aggregations of eagle rays (*Myliobatis aquila*) in a single volcanic cave from the Azores (NE Atlantic). *XIth European Congress of Ichthyology,* Tallinn, Estonia. Abstract Volume:129–130.

Başusta A., Başusta N., Sulikowski JA., Driggers WB., Demirhan SA., Çiçek E. 2012. Length-weight relationships for nine species of batoids from the Iskenderun Bay, Turkey. *Journal of Applied Ichthyology* 28:850–851.

Biais G., Hennache C., Stephan E., Delamare A. 2014. Mark-recapture abundance estimate of undulate ray in the Bay of Biscay. Working Document WGEF 2014. Lisbon, ICES CM, 10 pp.

Borges TC., Olim S., Erzini K. 2003. Weight–length relationships for fish species discarded in commercial fisheries of the Algarve (southern Portugal). *Journal* of *Applied Ichthyology* 19:394–396.

Cabral H., Catarino AI., Figueiredo J., Garcia J., Henriques M. 2003. Feeding ecology, age, growth and sexual cycle of the Portuguese sole, *Synaptura lusitanica*. *Journal of the Marine Biological Association of the United Kingdom* 83:613–618.

Carvalho N., Afonso P., Santos RS. 2003. The haremic mating system and mate choice in the wide-eyed flounder, *Bothus podas*. *Environmental Biology of Fishes* 66:249–258.

Coelho R., Erzini K. 2002. Age and growth of the undulate ray *Raja undulata*, in the Algarve (southern Portugal). *Journal of the Marine Biological Association of the United Kingdom* 82:987–990.

Consalvo I., Scacco U., Romanelli M., Vacchi M. 2007. Comparative study on the reproductive biology of *Torpedo torpedo* (Linnaeus, 1758) and *T. marmorata* (Risso, 1810) in the central Mediterranean Sea. *Scientia Marina* 71:213–222.

Dinis MT. 1986. Quatre Soleidae de l'Estuaire du Tage: Reproduction et Croissance: Essai d'Élevage de *Solea senegalensis* Kaup 1858. D. Phil. Thesis, Université de Bretagne Occidentale.

Dorel D. 1986. Poissons de l’Atlantique Nord-Est: relations taille-poids. Institut Français de Recherche pour L'Exploitation de la Mer. Nantes, Rapport IFREMER, 165 pp.

Dulčić J., Kraljević M. 1996. Weight-length relationships for 40 fish species in the eastern Adriatic (Croatian waters). *Fisheries Resources* 28:243–251.

Duman OV., Başusta N. 2013. Age and growth characteristics of marbled electric ray *Torpedo marmorata* (Risso 1810) inhabiting Iskenderun Bay, north-eastern Mediterranean Sea. *Turkish Journal of Fisheries and Aquatic Sciences* 13:541–549.

Filiz H., Bilge G. 2004. Length–weight relationships of 24 fish species from the North Aegean Sea, Turkey. *Journal* of *Applied Ichthyology* 20:431–432.

Fischer W., Schneider M., Bauchot ML. 1987. *Guide FAO d'identification des espèces pour les besoins de la pêche. Méditerranée et Mer Noire (Zone de Pêche 37)*. Volume II: Vertébrés. Rome: FAO.

Froese R., Pauly D. eds. 2017. FishBase. www.fishbase.org

García-Díaz MM., Tuset VM., González JA., Socorro J. 1997. Sex and reproductive aspects in *Serranus cabrilla* (Osteichthyes: Serranidae): macroscopic and histological approaches. *Marine Biology* 127:379−386.

Gharbi H, Ktari MH. 1981. Croissance des rougets en Tunisie. *Bulletin de l'Institut national scientifique et technique d'océanographie et de pêche de Salammbô* 8:5–40.

Gonçalves JMS., Bentes L., Lino PG., Ribeiro J., Canário AVM., Erzini K. 1997. Weight-length relationships for selected fish species of the small-scale demersal fisheries of the south and south-west coast of Portugal. *Fisheries Resources* 30:253–256.

Goosen AJJ., Smale MJ. 1997. A preliminary study of age and growth of the smooth-hound shark *Mustelus mustelus* (Triakidae). *South African Journal of Marine Science* 18:85–91.

Gordo LS., Neves A., Vieira AR., Paiva RB., Sequeira V. 2016. Age, growth and mortality of the comber *Serranus cabrilla* (Linnaeus, 1758) in the Eastern Atlantic. *Marine Biology Research* 12:656–662.

Hunter E., Buckley AA., Stewart C., Metcalfe JD. 2005. Migratory behaviour of the thornback ray, *Raja clavata*, in the southern North Sea. *Journal of the Marine Biological Association of the United Kingdom* 85:1095–1105.

ICES. 2012. Report of the Working Group on the Assessment of Demersal Stocks in the North Sea and Skagerrak (WGNSSK). Copenhagen, ICES CM 2012/ACOM:13, 1346 pp.

ICES, 2013. Report of the Working Group on Elasmobranch Fishes (WGEF). Lisbon, ICES CM 2013/ACOM:19, 637 pp.

İlkyaz AT., Metin G., Soykan O., Kınacıgil HT. 2017. Age, growth, and reproduction of Mediterranean scaldfish, *Arnoglossus laterna* (Actinopterygii: Pleuronectiformes: Bothidae), in the east-central Aegean Sea. *Acta Ichthyologica et Piscatoria* 47:53–61.

Ismen A., Ismen P., Basusta N. 2004. Age, Growth and Reproduction of Tub Gurnard (*Chelidonichthys lucerna* L. 1758) in the Bay of Iskenderun in the Eastern Mediterranean. *Turkish Journal of Veterinary and Animal Sciences* 28:289–295.

Ismen A., Ozen O., Altinagac U., Ozekinci U., Ayaz A. 2007. Weight–length relationships of 63 fish species in Saros Bay, Turkey. *Journal* of *Applied Ichthyology* 23:707–708.

Jrad LB., Fehri-Bedoui R., Slama SB., Hassine OKB., 2010. Reproduction et régime alimentaire de *Trigloporus lastoviza* (Triglidae) dans le golfe de Tunis (Méditerranée occidentale). *Cybium* 34:353–65.

Kadri H., Marouani S., Bradai MN., Bouain A., Morize E. 2014a. Age, Growth, Mortality, Longevity and Reproductive Biology of the White Skate, *Rostroraja alba* (Chondrichthyes: Rajidae) of the Gulf of Gabes (Southern Tunisia, Central Mediterranean). *Turkish Journal of Fisheries and Aquatic Sciences* 14:193–204.

Kadri H., Marouani S., Bradai MN., Bouain A., Morize E. 2014b. Age, growth and length-weight relationship of the white skate, *Rostroraja alba* (Linnaeus, 1758) (Chondrichthyans: Rajidae), from the Gulf of Gabes (Tunisia, Central Mediterranean). *Journal of Coastal Life Medicine* 2:421–425.

Korta M., García D., Santurtún M., Goikoetxea N., Andonegi E., Murua H., Álvarez P., Cerviño S., Castro J., Murillas A. 2015. European hake (Merluccius merluccius) in the Northeast Atlantic Ocean. In: Arancibia H, ed. Hakes: Biology and Exploitation. New Jersey: Wiley-Blackwell.

Koutrakis ET., Tsikliras AC. 2003. Short communication. Length-weight relationships of fishes from three northern Aegean estuarine systems (Greece). *Journal* of *Applied Ichthyology* 19:258–260.

Martin LK., Cailliet GM. 1988. Age and growth determination of the Bat Ray, *Myliobatis californica* Gill, in central California. *Copeia* 3:762–773.

Mendes B., Fonseca P., Campos A. 2004. Weight–length relationships for 46 fish species of the Portuguese west coast. *Journal* of *Applied Ichthyology* 20:355–361.

Mennes F. 1985. Multi species assessment of fish stocks off the western Sahara region with emphasis on the family Sparidae. *Fishbyte* 3:5–10.

Morales-Nin B. 1991. Parametros biologicos del salmonete de roca *Mullus surmuletus* (L. 1758), en Mallorca. *Boletin del Instituto Español de Oceanografia* 7:139–147.

Morato T., Afonso P., Lourinho P., Barreiros JP., Santos RS., Nash RDM. 2001. Length–weight relationships for 21 coastal fish species of the Azores, north-eastern Atlantic. *Fisheries Resources* 50:297–302.

Morato T., Afonso P., Carvalho N., Lourinho P., Santos RS., Krug HM., Nash RDM. 2007. Growth, reproduction and recruitment patterns of the wide-eyed flounder, *Bothus podas* Delaroche Pisces: Bothidae), from the Azores. *Marine Biology Research* 3:403–411. DOI: 10.1080/17451000701712331.

Morey G., Moranta J., Massutí E., Grau A., Linde M., Riera F., Morales-Nin B. 2003. Weight–length relationships of littoral to lower slope fishes from the western Mediterranean. *Fisheries Resources* 62:89–96.

Moura T., Figueiredo I., Farias I., Serra-Pereira B., Coelho R., Erzini K., Neves A., Gordo LS. 2007. The use of caudal thorns for ageing *Raja undulata* from the Portuguese continental shelf in relation to its productive cycle. *Marine and Freshwater Research* 58:983–992.

Muñoz M., Hernández MR., Sàbat M., Casadevall M. 2003. Annual reproductive cycle and fecundity of *Aspitrigla obscura* (Teleostei, Triglidae). *Vie et Milieu* 53:123–129.

Muus BJ., Nielsen JG. 1999. *Sea fish*. Scandinavian Fishing Year Book. Hedehusene.

Pajuelo JG., Socorro J., González JÁ., Lorenzo JM., Pérez-Peñalvo JA., Martínez I., Hernández-Cruz CM. 2006. Life history of the red-banded seabream *Pagrus auriga* (Sparidae) from the coasts of the Canarian archipelago. *Journal* of *Applied Ichthyology* 22:430–436.

Palazón-Fernández JL., Arias AM., Sarasquete MC. 2001. Aspects of the reproductive biology of the toadfish *Halobatrachus didactylus* (Schneider, 1801) (Pisces: Batrachoididae). *Scientia Marina* 65: 131–138.

Palazón-Fernández JL., Potts JC., Manooch III CS., Sarasquete C. 2010. Age, growth and mortality of the toadfish, *Halobatrachus didactylus* (Schneider, 1801) (Pisces: Batrachoididae), in the Bay of Cádiz (southwestern Spain). *Scientia Marina* 74: 121–130.

Petrakis G., Stergiou KI. 1995. Weight-length relationships for 33 fish species in Greek waters. *Fisheries Resources* 21:465–469.

Piñeiro C., Saínza M. 2003. Age estimation, growth and maturity of the European hake (*Merluccius merluccius* Linnaeus, 1758) from the Iberian Atlantic waters. *ICES Journal of Marine Science* 60:1086–1101.

Quigley DTG. 1984. White skate, *Raja alba* Lacepede, 1803 (*R. marginata*, Lacep.) in Irish waters: a further record and review of Irish records. *Irish Naturalists' Journal* 21:217–220.

Santos MN., Gaspar MB., Vasconcelos P., Monteiro CC. 2002. Weight–length relationships for 50 selected fish species of the Algarve coast (southern Portugal). *Fisheries Resources* 59:289–295.

Serra-Pereira B., Figueiredo I., Farias I., Moura T., Gordo LS. 2008. Description of dermal denticles from the caudal region of *Raja clavata* and their use for the estimation of age and growth. *ICES Journal of Marine Science* 65:1701–1709.

Serra-Pereira B., Figueiredo I., Gordo LS. 2011. Maturation, fecundity, and spawning strategy of the thornback ray, *Raja clavata*: do reproductive characteristics vary regionally? *Marine Biology* 158:187–2197.

Smale MJ., Compagno LJV. 1997. Life history and diet of two southern African smoothhound sharks, *Mustelus mustelus* (Linnaeus, 1758) and *Mustelus palumbes* Smith, 1957 (Pisces: Triakidae). *South African Journal of Marine Science* 18:229–248.

Tanner SE., Reis-Santos P., Vasconcelos RP., Thorrold SR., Cabral HN. 2013. Population connectivity of *Solea solea* and *Solea senegalensis* over time. *Journal of Sea Research* 76:82–88.

Teixeira CM., Batista MI., Cabral HN. 2010. Diet, growth and reproduction of four flatfishes on the Portuguese coast. *Scientia Marina* 74:223–233. DOI: 10.3989/sci-mar.2010.74n2223.

Tsimenides N., Machias A., Kallianiotis A. 1992. Distribution patterns of triglids (Pisces: Triglidae) on the Cretan shelf (Greece), and their interspecific associations. *Fisheries Research* 15:83–103.

Valle C., Bayle JT., Ramos AA. 2003. Weight–length relationships for selected fish species of the western Mediterranean Sea. *Journal* of *Applied Ichthyology* 19:261–262.

Vallisneri M., Stagioni M., Montanini S., Tommasini S. 2011. Body size, sexual maturity and diet in *Chelidonichthys lucerna* (Osteichthyes: Triglidae) from the Adriatic Sea, north eastern Mediterranean. *Acta Adriatica* 51:141–148.

Vassilopoulou V., Papaconstantinou C. 1994. Age, growth and mortality of the spotted flounder (*Citharus linguatula* Linnaeus, 1758) in the Agean Sea. *Scientia Marina* 58:261–267.

Veiga P., Machado D., Almeida C., Bentes L., Monteiro P., Oliveira F., Ruano M., Erzini K., Gonçalves JMS. 2009. Weight-length relationships for 54 species of the Arade estuary, southern Portugal. *Journal* of *Applied Ichthyology* 25:493–496.

Vinagre C., Maguas C., Cabral HN., Costa MJ. 2011. Nekton migration and feed location in a coastal area - a stable isotope approach. *Estuarine, Coastal and Shelf Science* 91:544–550. DOI: 10.1016/j.ecss.2010.12.011.

Walker P., Howlett G., Millner R. 1997. Distribution, movement and stock structure of three ray species in the North Sea and eastern English Channel. *ICES Journal of Marine Science* 54:97–808.
